# Streptococcus cristatus reduces cariogenicity of saliva-derived microcosms under pH-dependent conditions

**DOI:** 10.1080/20002297.2025.2565450

**Published:** 2025-10-07

**Authors:** Yanling Cai, Lijing Wu, Bernd W. Brandt, Mark J. Buijs, Xi Wei, Hongyan Liu, Dongmei Deng

**Affiliations:** a Hospital of Stomatology, Guanghua School of Stomatology, Sun Yat-sen University & Guangdong Provincial Key Laboratory of Stomatology, Guangzhou, China; b Academic Centre for Dentistry Amsterdam, University of Amsterdam and Vrije Universiteit Amsterdam, Amsterdam, The Netherlands

**Keywords:** *Streptococcus cristatus*, probiotics, cariogenicity, saliva, microcosms, dental caries

## Abstract

**Background:**

The study aims to investigate *Streptococcus cristatus*, an oral commensal bacterium, as a probiotic for dental caries prevention by modulating the oral microbiome.

**Methods:**

Saliva from four healthy donors was used to establish 24-h microcosm biofilms in an *in vitro* 96-well peg model. The preformed biofilms were then exposed to biofilm medium containing 0.2% sucrose (BM), with or without *S. cristatus*. They were grown for 48 h under two conditions: a constant pH-neutral regime (BM supplemented with 76 mM K_2_HPO_4_ and 15 mM KH_2_PO_4_, pH 7.0) or cariogenic pH-cycling regime (8 h pH-neutral and 16 h in BM containing 100 mM acetic acid, pH 5.5). Phosphate and acetate buffers were used to control pH. After 72 h, the biofilms were analyzed for biomass, lactic acid production, hydrogen peroxide (HP) concentrations, and microbial composition via 16S rRNA gene sequencing.

**Results:**

*S. cristatus* successfully integrated into 24-h preformed microcosm biofilms derived from individual saliva. Under pH-neutral conditions, it reduced biofilm biomass and lactate production while increasing hydrogen peroxide (HP) generation in a donor-dependent manner. Conversely, under cariogenic pH-cycling conditions, these inhibitory effects on biomass and lactate production were consistent across all donors, although HP was undetectable. Microbiome analysis revealed that *S. cristatus* increased species richness and mitigated the compositional shifts caused by pH-cycling. This was achieved by inhibiting *Streptococcus salivarius/vestibularis* across all donors, while promoting *Streptococcus mitis* group and *Streptococcus anginosus* in a donor-dependent manner.

**Conclusions:**

*S. cristatus* represents a promising microbiome modulator with the potential to substantially mitigate the cariogenicity of oral microcosms.

## KEY MESSAGES

• *S. cristatus* integrates into pre-formed biofilms, reducing biomass and lactate production while boosting hydrogen peroxide.
• The pH-cycling condition (8 h pH 7.0 and 16 h pH 5.5) enhances the inhibitory effect of *S. cristatus* on biomass and lactate production, and this effect is consistent across all donors.
• *S. cristatus* increases species richness and counters pH-cycling-induced dysbiosis via selective regulation of streptococci.

## Introduction

Dental caries is one of the most prevalent diseases globally, affecting 43% of deciduous teeth and 29% of permanent teeth [1]. This highlights the urgent need for effective prevention and treatment strategies to mitigate its impact on global health. Dental caries results from an imbalance between demineralisation and remineralisation of dental hard tissues [2], driven by dysbiotic multi-species biofilms. Microbes within these biofilms ferment dietary carbohydrates, producing organic acids that lower the pH of the surrounding environment. In a healthy situation, the microbiome maintains symbiosis through effective pH buffering, allowing transient pH drops followed by rapid recovery to neutral pH [3]. However, under cariogenic conditions, such as frequent sugar consumption, excess acid production or impaired acid neutralisation occurs. This leads to a compositional shift in the microbiota towards a dysbiotic state characterised by the enrichment of acidogenic and aciduric bacterial species like *Streptococcus mutans* and *Lactobacilli*. This changes periods of local low pH, disrupting the balance between remineralisation and demineralisation, and ultimately promoting the development of dental caries [4]. This ecological plaque hypothesis underlines the critical role of maintaining a balanced and diverse oral microbiota in preventing caries [5].

Caries prevention strategies traditionally include reducing free sugar intake, removing biofilm mechanically through brushing and flossing, inhibiting biofilm chemically with agents such as chlorhexidine, and promoting remineralisation with fluoride [6]. While these approaches are well established, they do not fully address the role of a balanced oral microbiome in caries development, as highlighted by the ecological plaque hypothesis. With growing recognition of the importance of maintaining healthy oral microflora, the focus has shifted from broadly suppressing oral bacteria to modulating the microbiome by controlling the overgrowth of cariogenic bacteria while supporting microbial diversity and stability [6–8]. Thus, microbiome modulation strategies, including the use of probiotics, are now being considered novel and complementary approaches for caries prevention.

Probiotics are defined as ‘live microorganisms that, when administered in adequate amounts, confer a health benefit on the host’ [9]. Since their discovery, probiotics have been extensively studied, gaining widespread public acceptance and use [10,11]. For example, in Europe, up to 60% of healthcare providers and 80% of general practitioners have recommend probiotics for conditions related to bowel diseases, such as Antibiotic Associated Diarrhoea, infectious diarrhoea and abdominal pain [12,13]. As promising microbiome modulators [14], probiotics have been proposed for caries prevention by counteracting disease-associated bacteria, supporting pH balance, and promoting a healthy oral ecosystem [7,14].

While *in vitro* and *in vivo* studies have consistently shown that probiotics reduce caries-associated bacteria [15,16], clinical studies have yielded less favourable outcomes [17–19]. One possible reason for this discrepancy is that *in vitro* models often fail to capture the complexity of the oral microbiome. Many *in vitro* studies have evaluated probiotics against isolated bacterial cultures or simple biofilms containing one or a few cariogenic species [20–23], overlooking critical factors like microbial interaction, niche exclusion and colonisation resistance that influence probiotic integration and function in the oral environment. Only limited studies have examined the effects of probiotics in a saliva-derived microcosm biofilms which better represent complex microbial communities [24]. In a Perspective on the future development of probiotics for maintaining healthy gut microbiome, Suez et al. 2019 highlighted the need for more representative *in vitro* models to better simulate the interaction between probiotics and microbiome or host [10].

Previous studies have identified *Streptococcus oligofermentans–*recently reclassified under the same *Streptococcus cristatus* clade as *S. cristatus* [25]*–*as a promising oral microbiome modulator. Isolated mainly from the dental biofilm of caries-free individuals [26], it is considered part of the resident oral microbiota and exhibits low sugar fermentation capacity. Notably, it can covert lactate to hydrogen peroxide (HP) via lactate oxidase, which inhibits the growth and acid production of *S. mutans* biofilms and three-species biofilms [27,28]. This inhibitory effect is observed both in neutral pH environment and under cariogenic conditions with prolonged low pH. However, its potential to prevent caries has not been evaluated in oral microbiome.

This study aims to evaluate whether *S. cristatus* can modulate the microbial composition and function of saliva-derived microcosm biofilms grown under *in vitro* conditions of constant neutral pH and pH-cycling. The pH-cycling condition simulates cariogenic conditions, alternating between 8 h of neutral pH and 16 h at pH 5.5 to mimic the effects of sugar consumption. Microcosm biofilms were inoculated using saliva from four independent healthy donors, reflecting the donor-dependent variability in biofilm composition observed in previous studies [29].

## Materials and methods

### Bacterial strains and growth media

*Streptococcus cristatus* LMG22279 was used in this study and routinely maintained on Brain-Heart Infusion (BHI) agar at 37 °C. The biofilm growth medium (BM) contains 0.3% (wt/vol) yeast extract, 10 mmol/L (NH_4_)_2_SO_4_, 35 mmol/L NaCl, 2 mmol/L MgSO_4_·7H_2_O and is supplemented with filter-sterilised vitamins (0.04 mmol/L nicotinic acid, 0.1 mmol/L pyridoxine HCl, 0.01 mmol/L pantothenic acid, 1 mmol/L riboflavin, 0.3 mmol/L thiamine HCl, and 0.05 mmol/L D-biotin), amino acids (4 mmol/L L-glutamic acid, 1 mmol/L L-arginine HCl, 1.3 mmol/L L-cysteine HCl, and 0.1 mmol/L L-tryptophan) and 0.2% sucrose [20]. The pH of BM was adjusted to either 7.0 by adding 76 mM K_2_HPO_4_ and 15 mM KH_2_PO_4_ (BM7), or to 5.5 by adding 100 mM acetic acid (BM5).

### Saliva collection

Individual saliva samples were collected from four donors, comprising three females and one male, aged 28–50 years, who were systemically and orally healthy (i.e., no active caries, gingivitis, or periodontitis) based on self-reporting and had not used antibiotics 3 months prior to collection. Each donor was asked to refrain from oral hygiene for 12 h and abstained from food–drink intake for at least 2 h prior to collection. The unstimulated saliva of individual donor was collected on ice, diluted with 60% (vol/vol) of glycerol at 1:1 ratio, and stored separately at −80 °C. Each donor was notified the objectives of the study and has signed an informed consent. The study protocol was approved by the Medical Ethical Committee of the VU University Medical Centre Amsterdam (document number 2011/236).

### Saliva-derived microcosm biofilm formation

The microcosm biofilms were grown in a 96-well peg model [20]. This model consists of a standard 96-well microtiter plate and a lid with 96 polystyrene pegs that fit into the wells (Nunc™, Roskilde, Denmark), which allows active attachments of bacterial cells.

To form a biofilm, saliva from individual donor was diluted with fresh BM7 at a ratio of 1:20 and dispensed into a 96-well plate (200 μL/well), which was then covered with the lid containing pegs and incubated anaerobically (80% N_2_, 10% CO_2_, 10% H_2_) at 37 °C for 24 h. The pegs with 24-h preformed biofilms were washed in distilled water for 10 s to remove unattached bacterial cells and then transferred to a new 96-well plate containing 200 μL/well of fresh BM7 only (M group) or *S. cristatus* cultures.

To prepare *S. cristatus* cultures, a single colony was inoculated into BM7 and grown to stationary phase (16 h). The culture was then diluted in fresh BM7 to an OD600 of 0.7, corresponding to 5 × 10^7^CFU/mL. This *S. cristatus* suspension and a 10-fold dilution in BM7 were dispensed at 200 μL/well into a 96-well plate, resulting in two cell densities, 1 × 10^6^ CFU/well (lower density, MSc_L group) or 1 × 10^7^ CFU/well (higher density, MSc_H group).

**Figure 1.**
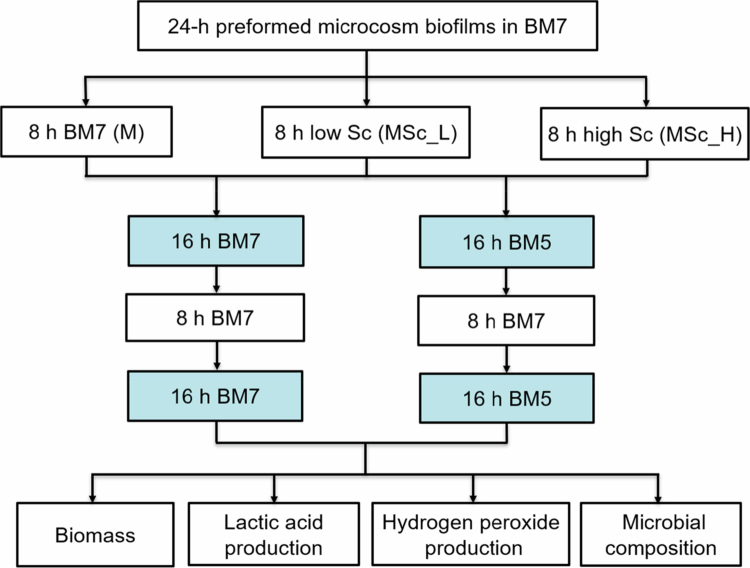
The experimental scheme of microcosm biofilm formation, growth conditions and outcome measurements. M: microcosm alone in the fresh BM7; Sc: *S. cristatus*; MSc_L: microcosm with a lower density of *S. cristatus* culture (1 × 10^6^ CFU/well) in the fresh BM7; MSc_H: microcosm with a higher density of *S. cristatus* culture (1 × 10^7^ CFU/well) in the fresh BM7; BM7: biofilm medium of pH 7.0; BM5: Biofilm medium of pH 5.5.

All biofilms were further grown under two pH conditions for another 48 h: constant neutral pH and pH-cycling conditions. For the pH-neutral condition, the biofilms were grown in BM7 with medium refreshments every 8 and 16 h. For the pH-cycling condition, the biofilms were grown in BM7 for 8 h and BM5 for 16 h. At 72-h, the biofilms on the pegs were rinsed with distilled water for 10 s to remove residual medium and unattached bacterial cells, then harvested for various assays, including biomass, lactic acid and HP production and microbial composition. The experimental scheme of biofilm formation and processing is illustrated in Figure 1. The study was performed with three biological replicates, each containing four parallel samples per group. For microbial composition analysis, a subset of samples (two parallel samples per group from two of the three biological replicates) were included.

### Biomass quantification

A crystal violet staining assay [30] was used to quantify the biomass of microcosm biofilms. In detail, the pegs with biofilms were inserted into 0.01% crystal violet solutions (200 μL/well) for 5 min, washed twice with distilled water to remove excess crystal violet, and then inserted into 2% sodium deoxycholate to destain for 5 min. The absorbance of the destaining solution was measured at 608 nm using a spectrophotometer (Spectramax Plus, Molecular Device, Sunnyvale, California, USA).

### Biofilm lactic acid and HP production

The pegs with microcosm biofilms were incubated in an assay buffer (200 μL/well) for 2 h at 37 °C, then an aliquot of biofilm spent solution was immediately used for HP measurement and the rest of the solution was stored at −20 °C for lactic acid quantification. The assay buffer is BM without yeast extract and sucrose, but with 1% glucose to stimulate lactic acid production. The yeast extract is removed to avoid bacterial growth during the 2-h incubation.

HP in biofilm spent solution was quantified by an enzymatic assay [27]. In detail, 50 μL of biofilm spent solution was mixed with 45 μL of solution containing 2.5 mM 4-aminoantipyrine (Sigma-Aldrich, St. Louis, MO, USA) and 0.17 M phenol in a 96-well plate for 5 min, followed by 5 μL of horseradish peroxidase (Sigma-Aldrich, St. Louis, MO, USA) with a final concentration of 640 mU/mL. After a 15-min incubation at room temperature, the absorbance was recorded at 510 nm in a spectrophotometer (Spectramax Plus, Molecular Device, Sunnyvale, California, USA). The HP concentration in each sample was calculated from a standard curve generated with a serial dilution of 30% HP (Sigma-Aldrich, St. Louis, MO, USA).

The lactic acid concentration in biofilm spent solution was measure with an in-house protocol [31]. In short, the biofilm spent solution was mixed with a solution comprising 2.0 M glycine, 1.6 M hydrazine sulphate, and 18 mg/mL NAD, and its absorbance at 340 nm was measured using a spectrophotometer (Spectramax Plus, Molecular Device, Sunnyvale, California, USA). Thereafter, 5 mg/mL L-lactate dehydrogenase was added to the mixture, incubated for 1 h, and the increased absorbance at 340 nm reflected the NADH formation through enzymatic conversion of L-lactate to pyruvate.

### 16S rRNA gene amplicon sequencing and sequencing data processing

The microbial composition of microcosm biofilms was analysed by 16S rRNA gene amplicon sequencing. To this end, the individual peg with biofilms was cut from the lid using a sterile scalpel and placed in 250 μL DNA-free Tris-EDTA buffer, followed by the standard genomic DNA (gDNA) extraction protocol in our laboratory [32]. The protocol includes phenol bead-beating to break up bacterial cell walls and gDNA purification by Mag mini kit (LGC Genomics, Berlin, Germany).

The V4 hypervariable region of the 16S rRNA genes were amplified with barcoded forward primer (5ʹ-GTGCCAGCMGCCGCGGTAA−3ʹ) and reverse primer (5ʹ-GGACTACHVGGGTWTCTAAT−3ʹ). The amplified products were pooled equimolarly, purified, and sequenced on the Illumina MiSeq platform at the VUmc Cancer Centre Amsterdam (Amsterdam, the Netherlands) to generate 251-bp paired-end reads. The raw data was quality-filtered and clustered into operational taxonomic units (OTUs) at 97% similarity [32]. The representative (most abundant) read of each OTU was assigned a taxonomy using the Ribosomal Database Project (RDP) classifier [33] and the SILVA version 132 database [34]. In order to identify the taxonomic names of a specific OTU, the representative sequence was aligned to the expanded Human Oral Microbiome Database version 15.2 (HOMD; http://www.homd.org) by blasting on the HOMD site using default parameters. The OTU table was subsampled at 5,200 reads per sample to achieve equal depths among samples.

### Quantification of *S. cristatus* in microcosm biofilms

A species-specific quantitative PCR (qPCR) was performed to quantify the amount of *S. cristatus* in the biofilm samples and saliva inocula, using the isolated gDNA as the template. *S. cristatus*-specific primers are: forward, 5ʹ-CATTTTACTGCATGGTAAGATG−3ʹ; reverse, 5ʹ-AAGGAGGTGATCCAGCC−3ʹ [35]. The reaction mixture containing SYBR Green (Thermo Fisher Scientific, Waltham, USA) was analysed using the LightCycler 480 II (Roche Diagnostics, Basel, Switzerland) as following: preincubation at 95 °C for 5 min and then 45 amplification cycles (denaturation at 95 °C for 10 s, annealing and extension at 57 °C for 20 s). The DNA concentration (pg/μL) of *S. cristatus* in each sample was calculated based on the standard curve derived from gDNA isolated from a pure *S. cristatus* culture.

#### Statistical analysis

Two-way analysis of variance (ANOVA) was performed with SPSS version 24 (IBM Corp., Armonk, NY, USA) to compare biomass formation, lactic acid production and HP production of three types of microcosm biofilms (M, MSc_L and MSc_H), using donors and biofilm growth conditions (pH-neutral or pH-cycling) as the independent variables, followed by a Bonferroni's post-hoc test for multiple comparisons. The data, including Shannon diversity index, relative abundance of OTUs, and gDNA concentrations of *S. cristatus*, did not satisfy the normal distribution or the homogeneity of variance, nonparametric test accompanied by Kruskal–Wallis was used for the analysis. Differences were considered of statistical significance if the *P-*value was less than 0.05.

For sequencing data analysis, the Shannon diversity index was calculated in PAST (Palaeontological Statistics) version 4.09, and statistical analysis was performed as described above. The OTU table obtained after data processing was log2-transformed and ordinated by principal component analysis (PCA), and the statistical differences in microbial composition were confirmed by one-way permutational multivariate analysis of variance (PERMANOVA), based on the Bray–Curtis similarity index and permutation of 9999. After filtering out OTUs with fewer than 10 reads, the linear discriminant analysis effect size (LEfSe) was applied to distinguish differentially abundant OTUs at two pH conditions, using the data of M group as the input data and to determine the OTUs which could differentiate M and MSc groups. The default settings were used. The redundancy analysis (RDA), using CANOCO version 5.10 [36], was selected to investigate the contributions of microcosm microbial composition to the biofilm characteristics (biomass, lactate and HP production).

## Results

In this study, all biofilms were supplied with 0.2% sucrose throughout the experimental phase. Although sucrose metabolism reduces pH, the duration and extent of low pH in the 96-well model are difficult to control, as they depend on factors such as nutrient availability and microbial composition. To address this, we established defined pH conditions to investigate the influence of *S. cristatus* on microbial composition. Biofilms were cultured in phosphate-buffered medium (pH 7.0) or acetate-buffered medium (pH 5.5), with medium exchange controlling the duration of exposure. Monitoring of spent media confirmed that these buffers maintained the intended pH-cycling conditions (8 h at pH 7.0 and 16 h at pH 5.5), even in the presence of sucrose.

### Biomass, lactic acid and HP production of microcosm biofilms

**Figure 2.**
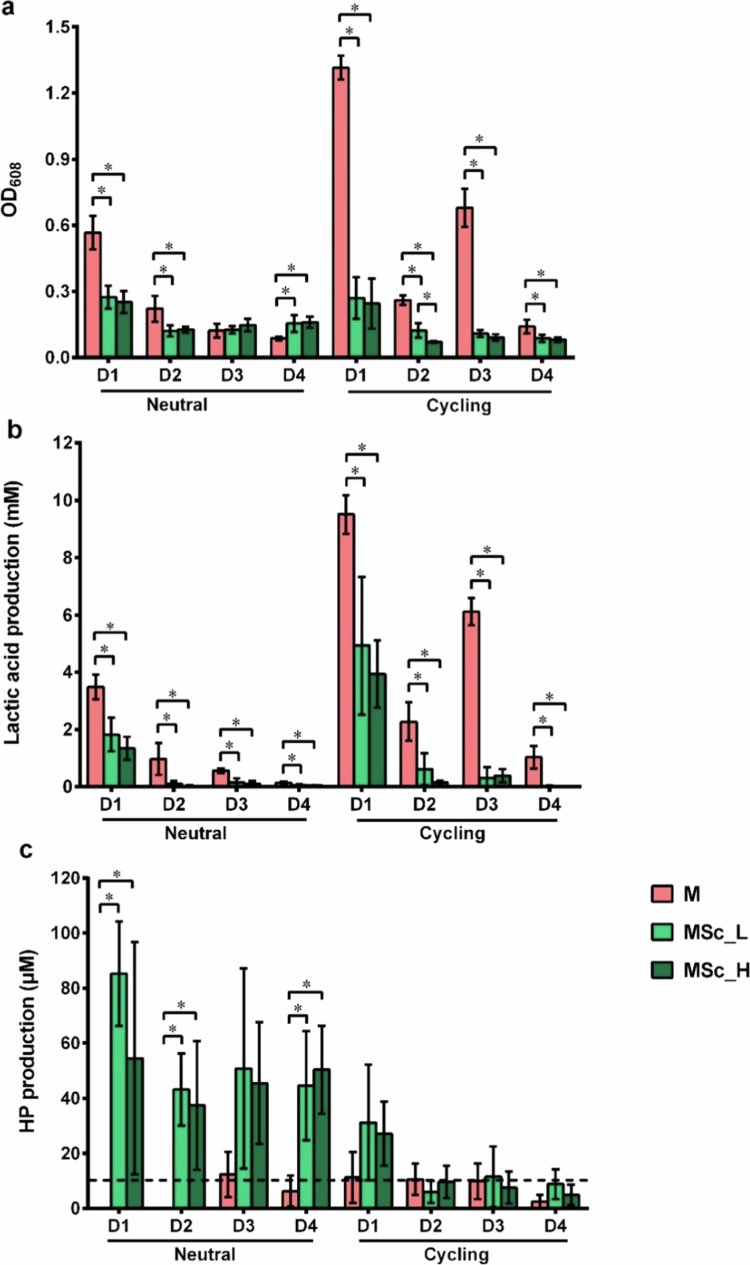
Biomass (a), lactic acid production (b) and hydrogen peroxide (HP) production (c) of microcosms, after 72 h, from four individual donors (D1–D4) with or without *S. cristatus* under either a constantly neutral pH (neutral) or a pH-cycling (cycling) condition. The dash line indicates the detection limit. * indicates the significant differences of biomass, lactic acid or HP production among the three microcosms for each donor in the same pH condition, *p < *0.05. D1: donor 1; D2: donor 2; D3: donor 3; D4: donor 4.

Figure 2 presents biomass (a), lactic acid (b) and HP (c) production of 48-h biofilms following the addition of *S. cristatus* to 24-h preformed microcosm biofilms. The biomass (OD_608_) in the microcosm alone (M) group was clearly donor-dependent. Biofilms grown under pH-cycling conditions exhibited significantly higher biomass in three out of four donors (donors 1, 3, and 4) compared to those grown under neutral pH conditions (Figure S1a). The addition of *S. cristatus*, whether at lower or higher density, altered the biomass in a manner dependent on both donor and the pH growth-conditions (Figure 2a). Under pH-neutral conditions, the addition of *S. cristatus* significantly reduced the biomass in biofilms from donors 1 and 2 but either did not affect or significantly increased the biomass in biofilms from donor 3 and 4. In contrast, under pH-cycling conditions, *S. cristatus* addition significantly reduced biomass in all biofilms, regardless of donor. Notably, the initial density of *S. cristatus* had no effect on the biomass outcome under any condition.

The lactic acid production followed a similar pattern to biomass (Figure 2b). Biofilms in the M group showed donor-dependent variations in lactic acid production (Figure S1b). Biofilms grown under pH-cycling condition produced higher amount of lactic acid compared to those grown under neutral condition. The addition of *S. cristatus* consistently reduced lactic acid production in all biofilms, irrespective of donor or pH condition. Similar to biomass, the initial density of *S. cristatus* had no impact on lactic acid production outcomes.

HP production was marginally detectable in the M group biofilms (Figure 2c, Figure S1c). The addition of *S. cristatus* significantly increased HP production under neutral pH conditions for most donors, with the exception of donor 3. In contrast, under pH-cycling conditions, HP concentrations were generally at or below the detection limit for all donors. Similar to biomass and lactic acid production, the initial density of *S. cristatus* had no significant effect on HP production under any condition.

#### Taxonomic profiles of the microcosms and the amount of *S. cristatus*

**Figure 3.**
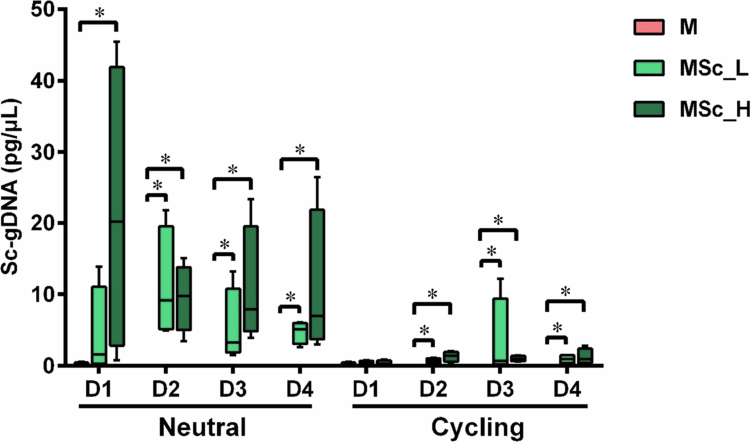
The gDNA concentrations of *S. cristatus* in the microcosms with or without added *S. cristatus* under either a constantly neutral pH (neutral) or a pH-cycling (cycling) condition. * indicates the significant differences of gDNA concentrations among the three microcosms in the same pH condition, *p < * 0.05.

Figure S2 provides an overview of the relative abundance of top 10 OTUs in the 72-h-old microcosm biofilms. *Streptococcus salivarius/vestibularis* (OTU1) and *S. cristatus* were the dominant species, together accounting for more than 60% of the total abundance. The representative sequence of OTU133 was identical to that of the spiked *S. cristatus* strain LMG22279. Figure 3 illustrates the presence of *S. cristatus* in the microcosm biofilms, as determined by qPCR. Both sequencing data and qPCR results showed that the relative abundance and quantity of *S. cristatus* significantly increased in the biofilms exposed to *S. cristatus*. However, the extent of the increase varied depending on the donor, pH condition, and detection method used. In the M group, biofilms contained no or very low amounts of *S. cristatus*.

#### Diversity of the microcosm biofilms

**Figure 4.**
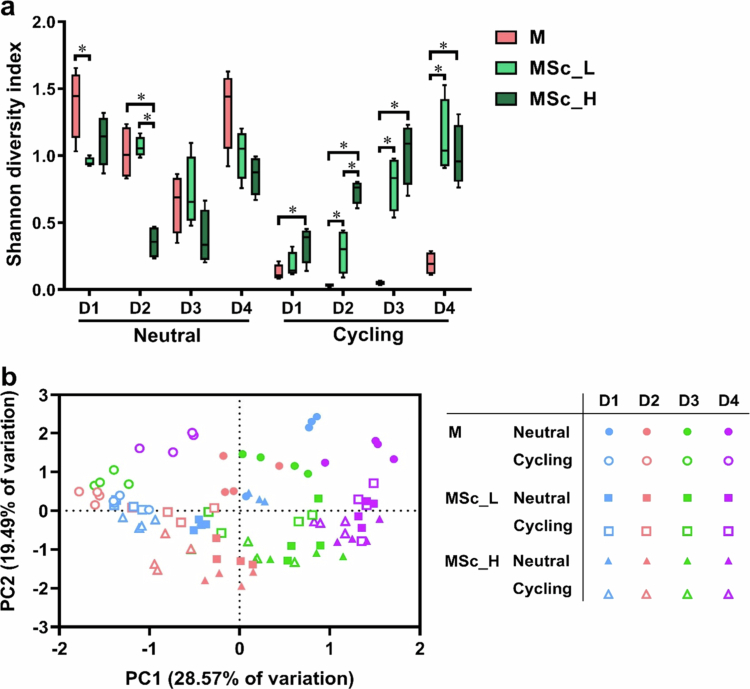
Differences in the microcosms with or without *S. cristatus* under either a constantly neutral pH (neutral) or a pH-cycling (cycling) condition. (a) The Shannon diversity index used to analyse *α*-diversity showed the microbial diversity of the different microcosm biofilms. (b) The principal component analysis (PCA) plot shows the ordination of the microbial profiles of the different microcosm biofilms. * indicates the significant differences among the three microcosms in each donor, *p < *0.05.

Figure 4 presents the Shannon Diversity Index (*α*-diversity) of various microcosm biofilms and a principal component analysis (PCA) plot based on their microbial profiles.

The Shannon index of the microcosm alone group (M) was significantly lower under pH-cycling conditions compared to pH-neutral conditions. Interestingly, the incorporation of *S. cristatus* affected the Shannon index differently depending on the growth pH condition: it either had no significant effect or decreased diversity under neutral pH conditions, whereas it increased the diversity under pH-cycling condition (Figure 4a). Notably, the average Shannon diversity index of the saliva inoculum from four donors was 2.8 ± 0.2, much higher than that observed in any of the biofilm communities.

The PCA plot (Figure 4b) revealed a clear separation along the PC2 axis between M biofilms and those containing *S. cristatus*. Additionally, the biofilms were also separated along the PC1 axis based on growth pH conditions. Incorporation of *S. cristatus* shifted the microcosm community along the PC1 axis and counteracted the effect of the growth pH, such that no distinct clustering based on pH conditions was observed. These findings were further confirmed by one-way PERMANOVA analysis (on pH conditions and biofilms) using the Bray-Curtis similarity index (Tables 1 and 2), which revealed considerable significant differences could be observed between M group biofilms and those containing *S. cristatus* under both neutral pH and pH-cycling conditions (*p *< 0.001). Notably, a pronounced significant difference between neutral pH and pH-cycling condition was observed only in the M group biofilms (*p *< 0.001).

**Table 1.** One-way PERMANOVA analysis of pairwise comparison in microcosm biofilms under each pH condition.

pH condition	Comparison	F value	*P* value
Neutral	M vs. MSc_L	**9.6**	**0.0003**
	M vs. MSc_H	**12.9**	**0.0003**
	MSc_L vs. MSc_H	1.9	0.3204
Cycling	M vs. MSc_L	**8.8**	**0.0003**
	M vs. MSc_H	13.7*	**0.0003**
	MSc_L vs. MSc_H	1.7	0.3843

* Comparisons with an F value greater than 4 and *p *< 0.001 are indicated in bold.

**Table 2.** One-way PERMANOVA analysis of pairwise comparison for pH conditions in each microcosm biofilms.

Microcosm biofilms	Comparison	F value	*P* value
M	Neutral vs. cycling	19.7*	**0.0001**
MSc_L	Neutral vs. cycling	3.1	0.0233
MSc_H	Neutral vs. cycling	2.9	0.0158

* Comparisons with an F value greater than 4 and *p *< 0.001 are indicated in bold.

#### Differentially abundant bacterial species in microcosms

Next, LEfSe was employed to identify differentially abundant OTUs following the addition of *S. cristatus* under various growth pH conditions. Since the initial density of *S. cristatus* did not significantly affect microbiota diversity, data from the MSc_L and MSc_H groups were combined into a single MSc group for comparison with the M group.

**Figure 5.**
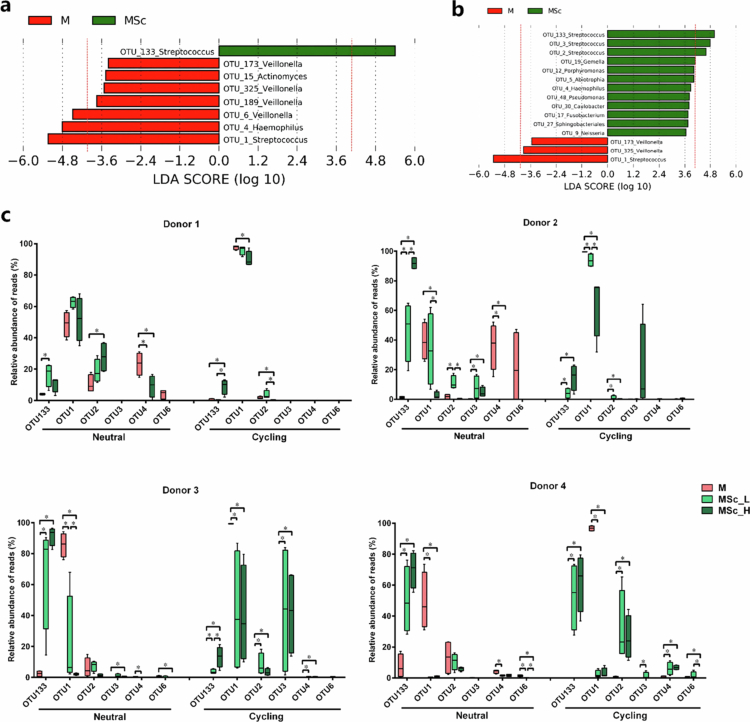
(a) The differentially abundant OTUs between the microcosms with and without *S. cristatus* under pH-neutral condition. The red dashed line indicates LDA = 4. (b) The differentially abundant OTUs between the microcosms with and without *S. cristatus* under pH-cycling condition. The red dashed line indicates LDA = 4. (c) Boxplots of the relative abundance of OTUs with an LDA score above 4.0 in microcosms with or without *S. cristatus* under pH-neutral (neutral) or a pH-cycling (cycling) condition by each donor. * indicates the significant differences of relative abundance among the three microcosms for each OTU, *p *<  0.05.

Figure 5a and b illustrate that the introduced *S. cristatus* (OTU133) was enriched in the MSc group, while *S. salivarius/vestibularis* (OTU1) remained dominant in the M group across both pH conditions. Other bacterial species exhibited pH-dependent variations: under neutral pH, *Haemophilus parainfluenzae* (OTU4) and *Veillonella parvula* (OTU6) were more abundant in the M group, whereas under pH-cycling conditions, *Streptococcus mitis* group (e.g. *Streptococcus infantis/mitis/oralis*, OTU2) and *Streptococcus anginosus* (OTU3) were prominent in the MSc group. OTUs with a linear discriminant analysis (LDA) score above 4.0 were plotted by donor, illustrating these shifts in Figure 5c.

The relative abundance of major OTUs exhibited donor-specific variations. Under neutral pH conditions in the M group, OTU1 and OTU4 together accounted for 73% of the microbiota in donor 1. In donor 2, OTU1, OTU4, and OTU6 collectively made up 96% of the microbiota, whereas in donor 3, OTU1 alone dominated at 86%. In donor 4, OTU1 and OTU2 together comprised 60% of the microbiota. Under pH-cycling conditions in the M group, OTU1 emerged as the predominant species across all donors. The addition of *S. cristatus* caused distinct shifts in microbiota composition compared to the M group, particularly under pH-cycling conditions, in a donor-dependent manner. While the relative abundance of OTU1 significantly decreased and OTU133 was markedly enriched across all donors, the relative abundance of OTU2 increased significantly in donors 3 and 4 and that of OTU3 increased in donor 3.

##### Microcosm biofilm characteristics and contributing bacterial species

**Figure 6.**
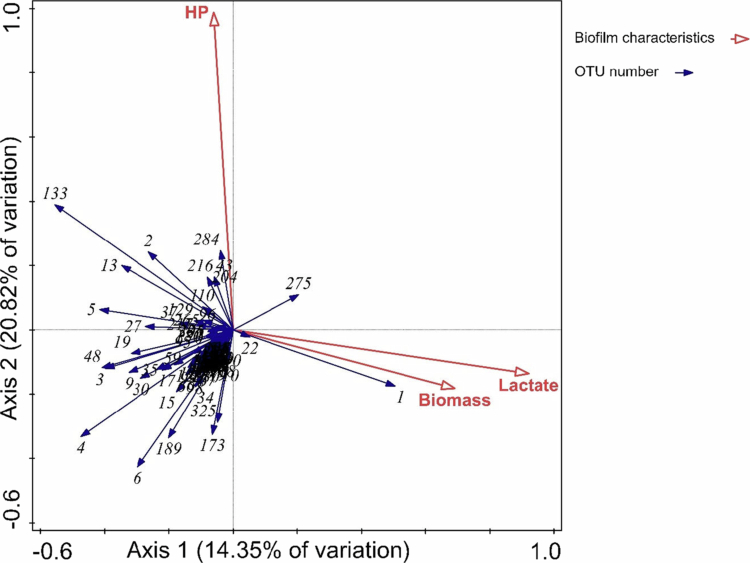
The ordination biplot of biofilm characteristics (biomass, lactate and HP production) and microbial composition by redundancy analysis (RDA). Axis 1 and axis 2 explained 14.35 and 20.82% of the variation, respectively. Based on the direction and the length of the projection point of OTUs arrow-tips lying in the arrows of the biofilm characteristics, OTU133 (*S. cristatus*), OTU2 (*S. mitis* group), and OTU284 (*Hydrogenobacter* sp. T−8) were the top 3 OTUs contributed to HP production, whereas OTU1 (*S. salivarius/vestibularis*) was the predominant OTU contributed to biomass and lactate production. The projection point lying in the same direction indicates a positive correlation, while the projection point lying in the opposite direction indicates a negative correlation. The farther a projection point falls in the direction indicated by the arrow, the higher the correlation.

Furthermore, the redundancy analysis (RDA) was performed to illustrate the contributions of bacterial species in microcosm biofilms to measured biofilm parameters, including biomass, lactate and HP productions. Axis 1 and 2 explained 35.2% of the variation in the relationship between OTU abundance and biofilm characteristics (Figure 6). The analysis indicated that the relative abundance of OTU1 was positively correlated with biomass and lactate production, while the relative abundance of OTU133 and OTU2 were positively correlated with HP production. In addition, OTU133 showed a negative correlation with biomass and lactate production, as evidenced by the projection point of its arrow-tip in the direction opposite to those of biomass and lactate in the RDA plot.

## Discussion

Oral probiotics may function as caries preventive agents by modulating dysbiotic oral microbiota [37–39]. In this study, we presented a potent probiotic candidate *S. cristatus*, which was capable of incorporating into a 24-h preformed saliva-derived microcosm biofilm. Following incorporation, *S. cristatus* effectively inhibited both biomass accumulation and lactic acid production within the biofilms, particularly under prolonged low pH conditions across all donors. These inhibitory effects are likely associated with changes in *Streptococcus* taxa, including a decrease in the relative abundance of tax related to *S. salivarius*/*vestibularis* and increase in those related to *S. mitis* group and *S. anginosus*. The findings strengthen the growing body of evidence [20,28,40], supporting *S. cristatus* as a potential microbiome modulator capable of mitigate the cariogenicity of oral microcosms.

Several important factors can influence the microbiome-modulating efficacy of an oral probiotic strain. The first factor is whether the probiotic strain is able to colonise the oral microbiome. A stable microbiota often resists colonisation by exogenous bacteria [41,42]. For instance, intestinal probiotic *Lactobacillus salivarius*, *Lactobacillus casei* and *Lactobacillus rhamnosus* GG failed to incorporate into or persist within saliva-derived biofilms, regardless of whether the biofilms had been pre-established for 24 or 48 h [43,44], highlighting the challenges of applying such strains for oral health applications. By contrast, indigenous oral probiotics show greater compatibility with the resident ecosystem. In this study, *S. cristatus*, a common oral species [26], was able to integrate into 24-h preformed biofilms under all tested conditions, indicating a possible ecological adaptation. Similar observations have been reported for another oral species, *Streptococcus dentisani* [45], further supporting the potential advantages of native probiotics. However, it is important to note that only young biofilms were examined in the present study. Further studies, including *in situ* biofilm models such as those used for *S. dentisani*, will be needed to determine whether *S. cristatus* can also incorporation into more mature biofilms under conditions that better reflect the natural oral environment, including saliva flow.

The second key factor is the unique characteristics of oral microbiome in each individual [46], which significantly influence probiotic efficacy [47,48]. Zmora et al. [48] identified ‘permissive’ individuals, who exhibited significant probiotics colonisation in their gastrointestinal (GI) mucosa, and ‘resistant’ individuals, who did not, emphasising the need for personalised precision probiotics. Another study demonstrated that the reduction of antibiotic resistance genes associated with probiotics was also person-specific [49]. In an *in vitro* biofilm model, Cieplik et al. [29] showed that biofilms grown from various oral niches retained patient-specific microbial signatures. Following this, we investigated the effect of *S. cristatus* on microcosm biofilms derived from four individual donors. Consistent with Cieplik et al.'s findings, biofilm biomass, lactic acid production, and major bacterial species were donor-dependent. Despite the donor variation in microcosm biofilms, *S. cristatus* reduced biomass (under pH-cycling conditions), biofilm lactic acid production, and the relative abundance of *S. salivarius/vestibularis* (under pH-cycling conditions) across all four donors, suggesting a donor-independent anti-caries effect of *S. cristatus*.

The third key factor is the environment in which the probiotics function. Research indicated that oral probiotics performed suboptimally under highly cariogenic conditions [22,50,51]. In this study, we established two distinct biofilm growth conditions: a constant pH-neutral regime, maintained by a buffered medium to prevent pH drop. Although such conditions do not occur *in vivo*, this regime represents a best-case scenario, resembling an environment with effective salivary buffering that restores pH toward neutrality; a pH-cycling regime characterised by prolonged low pH (5.5) to mimic a cariogenic environment. Monitoring the pH of biofilm media during experiments confirmed the validity of these designed conditions. Our results demonstrated that the addition of *S. cristatus* mitigated the composition shift induced by the pH-cycling environment, reduced differences between biofilms grown under pH-cycling and pH-neutral conditions, indicating that it could function effectively under cariogenic conditions.

In this study, we used RDA to infer how microbial interactions influence biofilm behaviour by linking species composition to functional outcomes. Our results suggest that *S. cristatus*, a HP producer [20], modulates the biofilms likely through HP-mediated interactions: it may inhibit lactic acid producers such as *S. salivarius* [52,53] while supporting the growth of other HP producers, likely members of the *S. mitis* group [54,55]. Although the exact species involved cannot be pinpointed due to sequencing resolution limitations, the findings indicate that *S. cristatus* influences biofilm composition by shifting the balance between lactic acid producers and HP producers. Further studies are needed to clarify the precise mechanisms by which *S. cristatus* exerts these modulatory effects.

Although this study employed complex saliva-derived microcosm biofilms and varied pH-regimes to investigate the biofilm modulation effects of *S. cristatus*, the model used has several limitations: 1. Species richness was much lower than in the original saliva inoculum, likely because the biofilm medium favoured some species while limiting others. Previous studies have shown that nutrient-poor media can reduce biofilm diversity and alter community composition [56]; 2. The cariogenic bacterium *S. mutans* was absent in all donor saliva and microcosm biofilms. Previous studies have demonstrated *S. cristatus* inhibited *S. mutans* in a two-species model [20], but it is unclear whether similar effects occur when *S. mutans* is present in a more complex community; 3. The substratum was polystyrene rather than enamel or dentin; 4. pH regulation relied on phosphate and acetate buffers. Although both buffers are naturally present in the oral cavity and sufficient to maintain the designed pH, the actual pH dynamics in the biofilm are more complex, involving salivary flow, salivary buffering and mineral buffering from enamel and dentin. Nevertheless, the data from this study provide a foundation for further investigation, supporting the validation of *S. cristatus'* effects on the oral microbiome in more realistic model, such as *in situ* models.

## Conclusions

Within limitations of this study, our data demonstrate that the *S. cristatus* successfully incorporated into pre-formed biofilms and inhibited biomass and lactic acid production, particularly under prolonged low pH conditions. This effect was achieved by preventing oral microbiota from shifting towards dysbiotic biofilms. These findings suggest that *S. cristatus* could serve as a potent microbiome modulator for caries prevention.

## Supplementary Material

Supplementary material **Figure S1.**Biomass **(a)**, lactic acid production **(b)** and hydrogen peroxide (HP) production **(c)** of 72-h microcosm biofilms without *S. cristatus*(M group), from 4 individual donors (D1-D4) under either a constantly neutral pH (Neutral) or a pH-cycling (Cycling) condition. The dash line indicates the detection limit. * indicates the significant differences of biomass, lactic acid or HP production between the microcosms in two pH conditions for each donor, *p <* 0.05. D1: donor 1; D2: donor 2; D3: donor 3; D4: donor 4.
**Figure S2.** Relative abundance (average of reads) of top 10 most abundant OTUs (remaining OTUs are grouped as “others”) in the 72-h microcosms with or without *S. cristatus* under either a constantly neutral pH (Neutral) or a pH-cycling (Cycling) condition.

## Data Availability

The datasets used or analysed during the current study are available from the corresponding authors on reasonable request.
