# Supplementary material for: Streptococcus cristatus reduces cariogenicity of saliva-derived microcosms under pH-dependent conditions
Source: J Oral Microbiol. 2025 Oct 7;17(1):2565450. doi: 10.1080/20002297.2025.2565450 (PMC12507114; doi:10.1080/20002297.2025.2565450)
Supplement: Supplementary material — Figure S1.Biomass (a), lactic acid production (b) and hydrogen peroxide (HP) production (c) of 72-h microcosm biofilms without S. cristatus(M group), from 4 individual donors (D1-D4) under either a constantly neutral pH (Neutral) or a pH-cycling (Cycling) condition. The dash line indicates the detection limit. * indicates the significant differences of biomass, lactic acid or HP production between the microcosms in two pH conditions for each donor, p < 0.05. D1: donor 1; D2: donor 2; D3: donor 3; D4: donor 4. Figure S2. Relative abundance (average of reads) of top 10 most abundant OTUs (remaining OTUs are grouped as “others”) in the 72-h microcosms with or without S. cristatus under either a constantly neutral pH (Neutral) or a pH-cycling (Cycling) condition. [file ZJOM_A_2565450_SM3070.docx]

**Supplementary material**

**
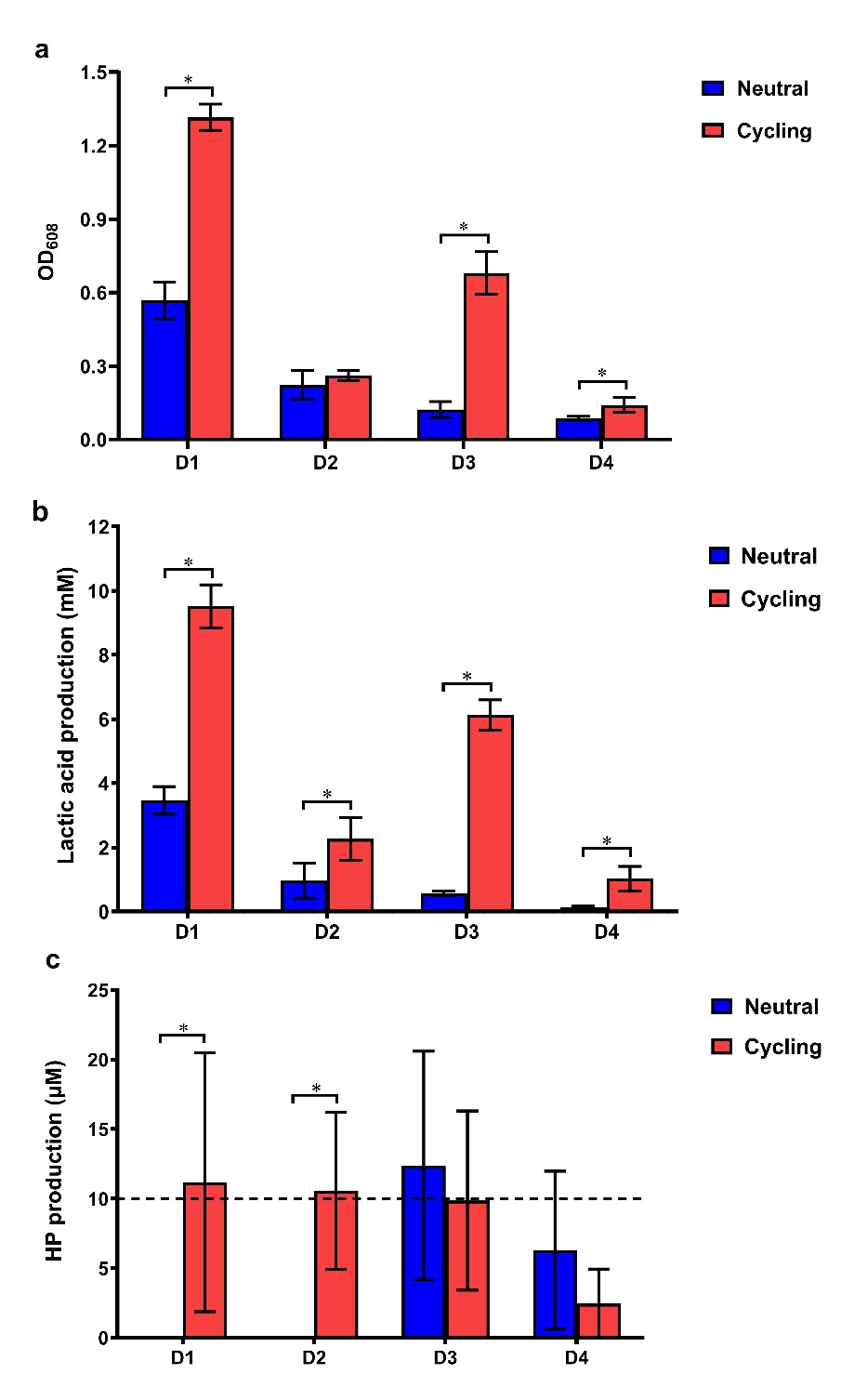
**

**Figure S1.** Biomass **(a)**, lactic acid production **(b)** and hydrogen peroxide (HP) production **(c)** of 72-h microcosm biofilms without *S. cristatus* (M group), from 4 individual donors (D1-D4) under either a constantly neutral pH (Neutral) or a pH-cycling (Cycling) condition. The dash line indicates the detection limit. * indicates the significant differences of biomass, lactic acid or HP production between the microcosms in two pH conditions for each donor, *p <* 0.05. D1: donor 1; D2: donor 2; D3: donor 3; D4: donor 4.

**
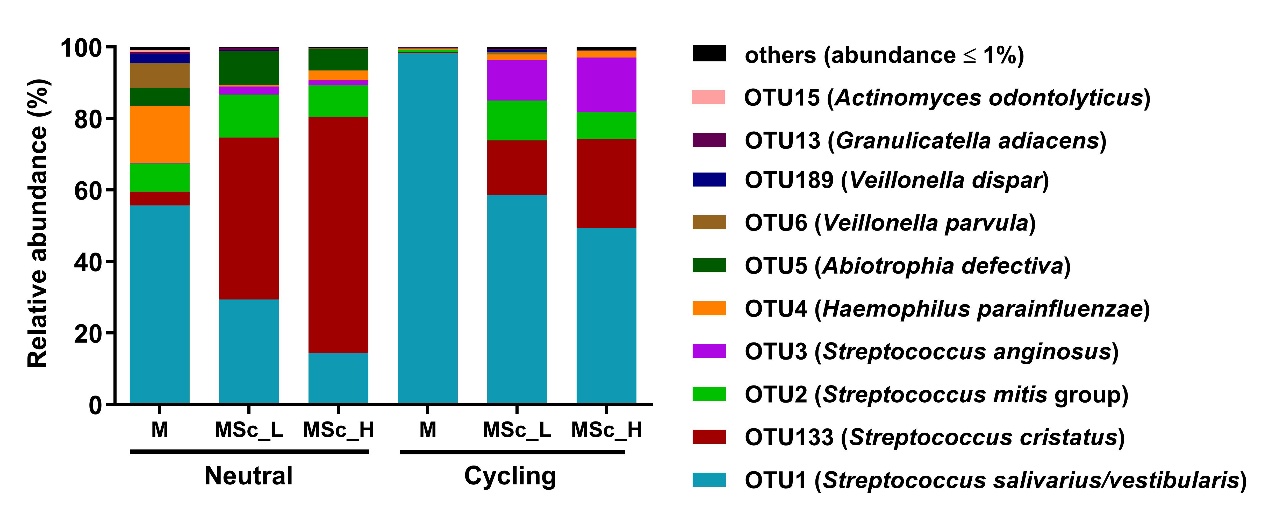
**

**Figure S2.** Relative abundance (average of reads) of top 10 most abundant OTUs (remaining OTUs are grouped as “others”) in the 72-h microcosms with or without *S. cristatus* under either a constantly neutral pH (Neutral) or a pH-cycling (Cycling) condition.
